# The photoelastic coefficient ${P}_{12}$ of H^+^ implanted GaAs as a function of defect density

**DOI:** 10.1038/s41598-017-14903-x

**Published:** 2017-11-09

**Authors:** Andrey Baydin, Halina Krzyzanowska, Rustam Gatamov, Joy Garnett, Norman Tolk

**Affiliations:** 1 Department of Physics and Astronomy, Vanderbilt University, Nashville, TN 37235 USA; 2 Institute of Physics, Maria Curie-Sklodowska University, Pl. M. Curie-Sklodowskiej 1, 20-031 Lublin, Poland; 3 Interdisciplinary Materials Science Program, Vanderbilt University, Nashville, TN 37235 USA; 4 Department of Life and Physical Sciences, Fisk University, Nashville, TN 37208 USA

## Abstract

The photoelastic phenomenon has been widely investigated as a fundamental elastooptical property of solids. This effect has been applied extensively to study stress distribution in lattice-mismatched semiconductor heterostructures. GaAs based optoelectronic devices (e.g. solar cells, modulators, detectors, and diodes) used in space probes are subject to damage arising from energetic proton (H^+^) irradiation. For that reason, the effect of proton irradiation on photoelastic coefficients of GaAs is of primary importance to space applied optoelectronics. However, there yet remains a lack of systematic studies of energetic proton induced changes in the photoelastic properties of bulk GaAs. In this work, the H^+^ energy and fluence chosen for GaAs implantation are similar to that of protons originating from the radiation belts and solar flares. We present the depth-dependent photoelastic coefficient ${P}_{12}$ profile in non-annealed H^+^ implanted GaAs obtained from the analysis of the time-domain Brillouin scattering spectra. The depth-dependent profiles are found to be broader than the defect distribution profiles predicted by Monte Carlo simulations. This fact indicates that the changes in photoelastic coefficient ${P}_{12}$ depend nonlinearly on the defect concentrations created by the hydrogen implantation. These studies provide insight into the spatial extent to which defects influence photoelastic properties of GaAs.

## Introduction

The photoelastic effect describes the coupling between light and sound in terms of the overall intensity and polarization properties of light scattering^1^. This effect has been applied to study stress distribution in semiconductor systems and lattice-mismatched semiconductor heterostructures. Its practical importance has been found in many optoelectronic devices such as light modulators, deflectors, and switches^2^. The knowledge of the photoelastic tensor is crucial for the proper design of cavity optomechanical systems^3,4^. Gallium arsenide (GaAs) is a semiconductor of the utmost importance for optoelectronics. Due to its relatively large photoelastic coefficients^5^, it is used for optomechanical resonators^4^. However, it is necessary to understand the influence of defects on the photoelastic coefficients in solids for reliable device fabrication. Defects, the origin of disorder, can be introduced into a specimen in various ways, e.g. during either materials growth, device fabrication processes or operation in harsh environments. Determining specifics of the relationship between structural disorder and basic optical properties, such as the complex refractive index and the photoelastic coefficients, is the key to understand the behavior of materials that have some amount of disorder. Proton (H^+^) irradiation in space is well known to be responsible for the degradation of satellite’s on-board electronics due to radiation damage^6–8^. Thus, understanding the damage (vacancies, interstitials, and their related defects) created by hydrogen implantation is crucial for designing reliable devices for use in space.

In this paper, we report depth profile and defect density dependence of the relative changes in the photoelastic coefficient ${P}_{12}$ caused by H^+^ implantation in GaAs (100). The depth dependent profile is obtained using the time-domain Brillouin scattering (TDBS) technique. This technique is also known as picosecond ultrasonics or coherent acoustic phonon (CAP) spectroscopy. It has already been applied to study properties of intrinsic GaAs^9–14^. Other experimental techniques such as stress induced birefringence, Brillouin scattering, and ellipsometry under uniaxial stress can only provide averaged bulk values of the photoelastic coefficients. TDBS, on the other hand, has been widely used to access depth dependent material properties such as elastic and optical inhomogeneities in disordered films^15–17^ ion implantation induced modification of interfacial bonding^18^, sub-*μ* m textures in materials compressed at megabar pressures^19,20^ doping profiles^21^, distribution of stress^22^, imaging of grain microstructure^23^, and determination of laser-induced temperature gradients in liquids^24^. Recently, we applied this technique to determine depth profiles of the complex refractive index modification arising from H^+^ implantation in 4H-SiC^25^. Point defect concentration profiles and optical damage cross-sections were obtained in He^++^^26^ and Ne^++^^27^ implanted GaAs, respectively. The application of TDBS to He^++^ implanted diamond revealed fluence dependent changes in the complex refractive index and sign reversal of the photoelastic coefficient ${P}_{12}$^28^. To the best of our knowledge, there is no other non-destructive technique capable of measuring depth dependent changes in photoelastic coefficients with high resolution. In general, the field of ion implanted semiconductors suffers from a lack of knowledge of the dependence of photoelastic coefficients on defect density.

## Results and Discussion

### Experimental Spectra

**Figure 1:**
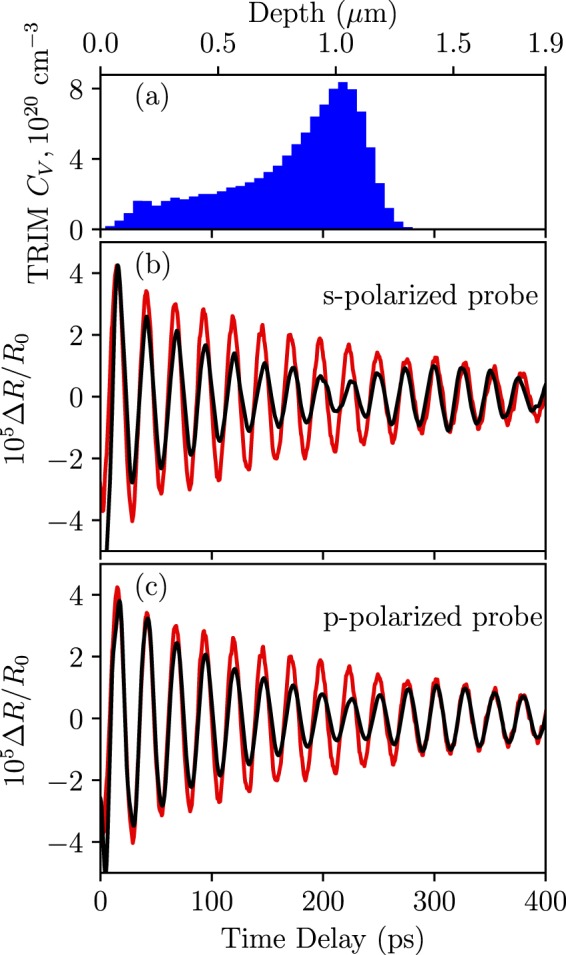
The damage-induced vacancy distribution as calculated by the TRIM code is shown in (**a**). Brillouin oscillations in the pump-probe reflectivity signal of the H^+^ implanted GaAs specimens for (**b)** s- and (**c**) p-polarized probe beam (in black). The probe wavelength is 880 nm. The implantation fluence is $3\,\times \,{10}^{15}$ cm^−2^. Red curves represent the corresponding signal for an unimplanted specimen.

Time-domain Brillouin scattering, also known as picosecond ultrasonics, is a pump-probe technique. Picosecond ultrasonics has been thoroughly reviewed by Matsuda *et al*.^29^. An incoming femtosecond pump pulse generates a coherent acoustic phonon wave which is a picosecond strain wave traversing the material at the speed of sound. To facilitate the generation of high amplitude coherent acoustic phonons, a thin metal film is typically deposited onto the material surface. For our experiments, a titanium layer of 20 nm was deposited using e-beam evaporation. The acoustic impedance mismatch between Ti and GaAs is negligible in that it ensures CAP wave transfer from Ti to GaAs without reflection at the interface. Generation of CAP waves in the metallic transducer can be classically explained by thermal expansion^30^. A time-delayed probe beam is then reflected both from the surface of the material and from the traveling CAP wave, giving rise to Brillouin oscillations due to interference between two reflected beams. The oscillation amplitude and frequency are dependent on material properties. Therefore, the damaged region in the ion implanted specimen will result in a different oscillatory signal compared to the unimplanted specimen. The Brillouin oscillations are always superimposed on the thermal response of the metallic transducer. In the following analysis, the thermal background has been subtracted out, leaving only the oscillatory part of the signal. Figure 1 shows Brillouin oscillations for unimplanted (red) and implanted (black) GaAs specimens at $3\,\times \,{10}^{15}$ cm^−2^ fluence for different probe polarizations. The damage-induced vacancy distribution calculated by the transport of ions in matter (TRIM) code^31^ is shown at the top of Fig. 1. The important observation to be derived from this data is that the oscillation amplitude decreases in the damaged region as indicated by the vacancy profile while the period and phase remain identical over the entire time (and thus, depth) window for both specimens. The reduction in oscillation amplitude cannot be attributed to the changes in the complex refractive index or speed of sound. Changes in the complex refractive index and speed of sound will result in the cumulative changes in the oscillation amplitude and period passed the damage region. It is seen in Fig. 1 that the oscillation amplitude of the implanted specimen becomes congruent with that of the unimplanted specimen passed the damaged region. Therefore, the modulation of the oscillation amplitude in the damaged region can be entirely attributed to the changes in the derivative terms of optical constants $\partial n/\partial \eta ,\partial \kappa /\partial \eta$^30^, and consequently to the photoelastic coefficients ${P}_{12}$ and ${P}_{11}$. The difference in the oscillation amplitude in the damaged region for s- and p-polarization of the probe beam arises from different photoelastic contributions to the oscillation amplitude (see Fig. 1). This observation is discussed in detail in the next section.

### Theoretical analysis

Following the derivation of transient reflectivity for a two layer system with oblique incident probe light by Matsuda and Wright^32^, the perturbation in dielectric constant ${\varepsilon }_{pe}(z,t)$ in isotropic material (such as GaAs) depends on the strain ${\eta }_{zz}(z,t)$ and photoelastic tensor components ${P}_{11}$ and ${P}_{12}$ (that are depth dependent in our case due to the damage arising from H^+^ implantation) as

1 $${\varepsilon }_{pe}(z,t)=(\begin{array}{ccc}{P}_{12}^{(j)}(z) & 0 & 0\\ 0 & {P}_{12}^{(j)}(z) & 0\\ 0 & 0 & {P}_{11}^{(j)}(z)\end{array}){\eta }_{zz}(z,t),$$

where index *j* indicates layer number. Any changes in the complex refractive index are negligible as discussed in the previous section, and thus its value is constant with respect to the depth coordinate, *z*. The speed of the CAP wave does not change in the implanted region because our data does not show a phase shift between the oscillatory signals corresponding to implanted and unimplanted specimens. Thus, the complex reflectance change for s- and p-polarized light is then given by^32^:

2 $$\begin{array}{ccc}\frac{\delta {r}^{(s)}}{{r}^{(s)}} & = & \frac{2{k}^{2}}{2{k}_{0}{a}_{0}^{(s)}{b}_{0}^{(s)}}[{\int }_{0}^{d}\eta ({z}^{^{\prime} },t){({a}_{1}^{(s)}{e}^{i{k}_{1}{z}^{^{\prime} }}+{b}_{1}^{(s)}{e}^{-i{k}_{1}{z}^{^{\prime} }})}^{2}d{z}^{^{\prime} }\\  &  & +{\int }_{0}^{{\rm{\infty }}}{P}_{12}^{(2)}({z}^{^{\prime} })\eta ({z}^{^{\prime} }+d,t){({a}_{2}^{(s)}{e}^{i{k}_{2}{z}^{^{\prime} }})}^{2}d{z}^{^{\prime} }\\  &  & +\,u(0,\,t)(1-{\varepsilon }_{1}){({a}_{1}^{(s)}+{b}_{1}^{(s)})}^{2}+u(d,t)({\varepsilon }_{1}-{\varepsilon }_{2}){({a}_{2}^{(s)})}^{2}],\end{array}$$

3 $$\begin{array}{ccc}\frac{\delta {r}^{(p)}}{{r}^{(p)}} & = & \frac{i}{2{k}_{0}{a}_{0}^{(p)}{b}_{0}^{(p)}}[\frac{{k}_{1}^{2}}{{\varepsilon }_{1}}{P}_{12}^{(1)}{\int }_{0}^{d}\eta ({z}^{^{\prime} },t){({a}_{1}^{(s)}{e}^{i{k}_{1}{z}^{^{\prime} }}+{b}_{1}^{(s)}{e}^{-i{k}_{1}{z}^{^{\prime} }})}^{2}d{z}^{^{\prime} }\\  &  & +\frac{{k}_{x}^{2}}{{\varepsilon }_{1}}{P}_{11}^{(1)}{\int }_{0}^{d}\eta ({z}^{^{\prime} },t){({a}_{1}^{(s)}{e}^{i{k}_{1}{z}^{^{\prime} }}-{b}_{1}^{(s)}{e}^{-i{k}_{1}{z}^{^{\prime} }})}^{2}d{z}^{^{\prime} }\\  &  & +{\int }_{0}^{{\rm{\infty }}}\frac{{k}_{2}^{2}{P}_{12}^{(2)}({z}^{^{\prime} })-{k}_{x}^{2}{P}_{11}^{(2)}({z}^{^{\prime} })}{{\varepsilon }_{2}}\eta ({z}^{^{\prime} }+d,t){({a}_{2}^{(p)}{e}^{i{k}_{2}{z}^{^{\prime} }})}^{2}d{z}^{^{\prime} }\\  &  & +u(0,\,t)(1-{\varepsilon }_{1})\{\frac{{k}_{1}^{2}}{{\varepsilon }_{1}}{({a}_{1}^{(p)}+{b}_{1}^{(p)})}^{2}-{k}_{x}^{2}{({a}_{1}^{(p)}-{b}_{1}^{(p)})}^{2}\}\\  &  & +u(d,t)({\varepsilon }_{1}-{\varepsilon }_{2})(\frac{{k}_{2}^{2}}{{\varepsilon }_{2}}-\frac{{k}_{x}^{2}}{{\varepsilon }_{1}}){({a}_{2}^{(s)})}^{2}],\end{array}$$

where ${r}^{(\mu )}={b}_{\mathrm{(0)}}^{(\mu )}/{a}_{\mathrm{(0)}}^{(\mu )}$ is the reflectance for the unperturbed (by the strain wave) sample, *d* is the thickness of the transducer layer, ${k}_{j}=\sqrt{{\varepsilon }_{j}{k}^{2}-{k}_{x}^{2}}$ is the wave vector in *j*-th medium, *k* is the wave vector in vacuum, ${a}_{j}$ and ${b}_{j}$ are the electric field amplitudes in *j*-th layer, *u* is the displacement, ${\varepsilon }_{1}$ and ${\varepsilon }_{2}$ are dielectric constants of the transducer and the substrate, respectively^32^. The first term in equation (2) and first two terms in equation (3) describe contribution to the reflectivity change when the strain wave is traveling through the transducer layer, once it leaves the layer, these terms vanish. We ignore any contribution from the static strain caused by elevated temperature of the transducer layer. Terms that include displacement of the surface and the interface, $u(z,t)={\int }_{-\infty }^{z}\eta (z^{\prime} ,t)dz^{\prime}$, also vanish when the strain wave is transmitted to GaAs. Therefore, we can rewrite equations (2) and (3) as following

4 $$\frac{\delta {r}^{(s)}}{{r}^{(s)}}=\frac{2{k}^{2}}{2{k}_{0}{a}_{0}^{(s)}{b}_{0}^{(s)}}{\int }_{0}^{{\rm{\infty }}}{P}_{12}^{(2)}({z}^{^{\prime} })\eta ({z}^{^{\prime} }+d,t){({a}_{2}^{(s)}{e}^{i{k}_{2}{z}^{^{\prime} }})}^{2}d{z}^{^{\prime} },$$

5 $$\frac{\delta {r}^{(p)}}{{r}^{(p)}}=\frac{i}{2{k}_{0}{a}_{0}^{(p)}{b}_{0}^{(p)}}{\int }_{0}^{{\rm{\infty }}}\frac{{k}_{2}^{2}{P}_{12}^{(2)}({z}^{^{\prime} })-{k}_{x}^{2}{P}_{11}^{(2)}({z}^{^{\prime} })}{{\varepsilon }_{2}}\eta ({z}^{^{\prime} }+d,t){({a}_{2}^{(p)}{e}^{i{k}_{2}{z}^{^{\prime} }})}^{2}d{z}^{^{\prime} }.$$

For the implanted specimen, we can make an assumption that the components of the photoelastic tensor are slowly varying functions and therefore can be assumed to be constant for the width of the strain pulse, which is estimated to be of the order of 30 nm for the Ti/GaAs structure. Thus, we can take ${P}_{12}(z)$ and ${P}_{11}(z)$ out of the integral

6 $$\frac{\delta {r}^{(s)}}{{r}^{(s)}}=\frac{2{k}^{2}}{2{k}_{0}{a}_{0}^{(s)}{b}_{0}^{(s)}}{P}_{12}^{(2)}({v}_{s}t){\int }_{0}^{{\rm{\infty }}}\eta ({z}^{^{\prime} }+d,t){({a}_{2}^{(s)}{e}^{i{k}_{2}{z}^{^{\prime} }})}^{2}d{z}^{^{\prime} },$$

7 $$\frac{\delta {r}^{(p)}}{{r}^{(p)}}=\frac{i}{2{k}_{0}{a}_{0}^{(p)}{b}_{0}^{(p)}}\frac{{k}_{2}^{2}{P}_{12}^{(2)}({v}_{s}t)-{k}_{x}^{2}{P}_{11}^{(2)}({v}_{s}t)}{{\varepsilon }_{2}}{\int }_{0}^{{\rm{\infty }}}\eta ({z}^{^{\prime} }+d,t){({a}_{2}^{(p)}{e}^{i{k}_{2}{z}^{^{\prime} }})}^{2}d{z}^{^{\prime} }.$$

If we write equations (6) and (7) for both implanted and unimplanted specimens, then subtract implanted from unimplanted and divide by unimplanted, we obtain

8 $$\frac{{(\delta {r}^{(s)}/{r}^{(s)})}_{U}-{(\delta {r}^{(s)}/{r}^{(s)})}_{I}}{{(\delta {r}^{(s)}/{r}^{(s)})}_{U}}=\frac{{[{P}_{12}^{(2)}]}_{U}-{[{P}_{12}^{(2)}({v}_{s}t)]}_{I}}{{[{P}_{12}^{(2)}]}_{U}}\equiv -\frac{{\rm{\Delta }}{P}_{12}}{{P}_{12}},$$

9 $$\frac{{(\delta {r}^{(p)}/{r}^{(p)})}_{U}-{(\delta {r}^{(p)}/{r}^{(p)})}_{I}}{{(\delta {r}^{(p)}/{r}^{(p)})}_{U}}=\frac{{[{P}_{eff}^{(2)}]}_{U}-{[{P}_{eff}^{(2)}({v}_{s}t)]}_{I}}{{[{P}_{eff}^{(2)}]}_{U}}\equiv -\frac{{\rm{\Delta }}{P}_{eff}}{{P}_{eff}},$$

where ${P}_{eff}^{(2)}=[{k}_{2}^{2}{P}_{12}^{(2)}-{k}_{x}^{2}{P}_{11}^{(2)}]\,/{\varepsilon }_{2}$, indices *U* and *I* represent unimplanted and implanted specimens, respectively.

### Depth dependence of the photoelastic coefficients

**Figure 2:**
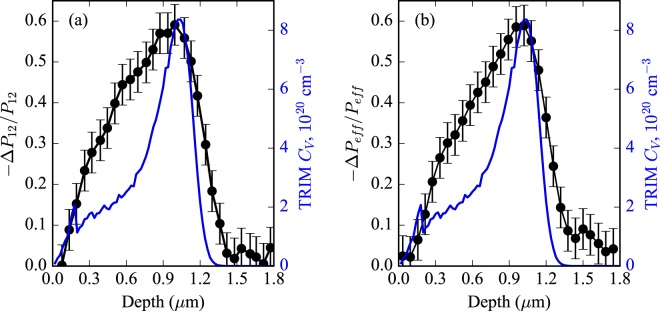
Depth dependent profiles of the relative changes in the photoelastic coefficients ${\rm{\Delta }}{P}_{12}/{P}_{12}$ (**a**) and ${\rm{\Delta }}{P}_{eff}/{P}_{eff}$ (**b**) of GaAs implanted at $3\,\times \,{10}^{15}$ cm^−2^ with 140 keV H^+^. The error bars were estimated from statistical analysis of a set of experimental spectra.

By processing the amplitudes of the Brillouin oscillations for implanted and unimplanted specimens according to equations (8) and (9), we obtain the relative changes in the photoelastic coefficients with respect to the depth for H^+^ implanted GaAs. As seen in Fig. 2, the profiles of the relative changes in the photoelastic coefficients show two regimes in depth: from 0 *μ*m to 0.2 *μ*m they follow the vacancy profile and from 0.2 *μ*m to 1 *μ*m they reveal different trend (it is broader) than that of the vacancy profile as obtained from the the TRIM code simulations. This fact indicates a nonlinear dependence of modified photoelastic coefficients on vacancy/defect concentration. The effect on the photoelastic properties due to ion implantation extends much further than the structural damage. The peak of the relative changes of both photoelastic coefficients ${\rm{\Delta }}{P}_{12}/{P}_{12}$ and ${\rm{\Delta }}{P}_{eff}/{P}_{eff}$ is about 60%. ${\rm{\Delta }}{P}_{11}$ has a factor ${k}_{x}^{2}$ in the definition of ${\rm{\Delta }}{P}_{eff}$ whereas ${\rm{\Delta }}{P}_{12}$ has a factor of ${k}_{2}^{2}$. In our case, ${k}_{x}^{2}\ll {k}_{2}^{2}$ that results in small contribution of ${P}_{11}$ to ${P}_{eff}$. Thus, we were not able to extract ${\rm{\Delta }}{P}_{11}/{P}_{11}$ from ${\rm{\Delta }}{P}_{eff}/{P}_{eff}$ because any difference between ${\rm{\Delta }}{P}_{12}/{P}_{12}$ and ${\rm{\Delta }}{P}_{eff}/{P}_{eff}$ are on the order of the noise present.

**Figure 3:**
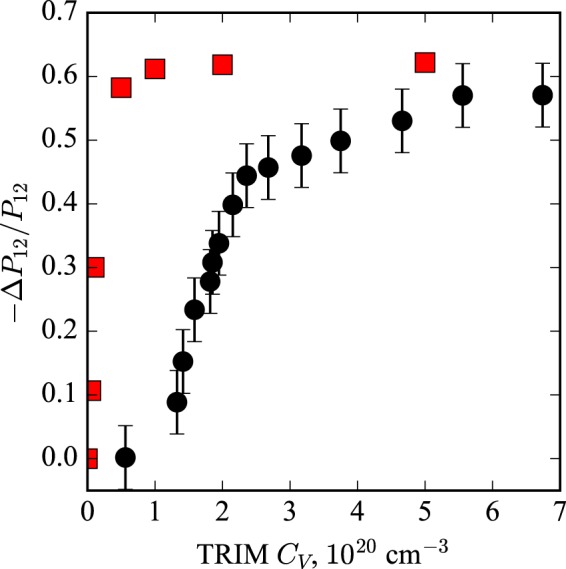
Black circles represent the relative changes in the photoelastic coefficient ${P}_{12}$ with respect to vacancy concentration. Red squares represent calculated values for the relative changes in the photoelastic coefficient ${P}_{12}$ as a function of vacancy concentration derived from a previous study^27^.

Figure 3 shows the dependence of the relative changes in the photoelastic coefficient ${P}_{12}$ with respect to the vacancy concentration. It is obtained by dividing the relative changes in the photoelastic coefficient ${P}_{12}$ by corresponding vacancy concentration as predicted by the TRIM code. As defect density (vacancy concentration) increases, the change in the photoelastic coefficient also increases towards its saturation value.

A. Steigerwald *et al*.^27^ have estimated optical constants (*n* and *κ*) and their derivatives ($\partial n/\partial E$ and $\partial \kappa /\partial E$) with respect to defect concentrations in disordered GaAs crystal using phenomenological band structure calculations. Their model assumes isolated, randomly placed point defects, which is an oversimplification of the clustered defect configurations one usually assumes with ion implantation damage. However, it has an advantage to study disordered systems at a low computational cost. The photoelastic coefficient ${P}_{12}$ is propotional to the strain derivatives of the optical constants as

10 $${P}_{12}\propto \sqrt{{[\frac{{\rm{\partial }}n}{{\rm{\partial }}E}]}^{2}+{[\frac{{\rm{\partial }}\kappa }{{\rm{\partial }}E}]}^{2}}\frac{{\rm{\partial }}E}{{\rm{\partial }}\eta },$$

where $\partial (n,\kappa )/\partial E$ is the rate of the change in the refractive index versus photon frequency, and $\partial E/\partial \eta$ is a deformation potential. Thus, by using equation (10) and the values of derivatives $\partial n/\partial E$, $\partial \kappa /\partial E$ from the ref.^27^, we obtain several theoretical data points for our range of vacancy concentrations. These points are presented in Fig. 3 as red squares. The relative changes in the photoelastic coefficient ${P}_{12}$ obtained by the simple phenomenological model^27^ follow a trend similar to the experimental data but the calculated model dependent changes in the photoelastic coefficient are overestimated at lower defect densities as seen in Fig. 3. This disagreement may be explained by the fact that the model is based on isolated point defects and does not account for any clustered defect configurations.

## Conclusion

In conclusion, we have demonstrated that TDBS can be applied to measure depth profiles of photoelastic coefficients in hydrogen ion bombarded GaAs. The method proposed here is suitable only for low fluences of implantation (low structural damage) because at higher implantation doses, changes in the complex index of refraction and sound velocity may occur. In the case when two or more quantities (refractive index, speed of sound, photoelastic coefficients) depend on a depth coordinate; a theory incorporating all depth dependent quantities such as developed by V. Gusev *et al*.^16^ should be applied. Experimental results for H^+^ implanted GaAs show that the implantation damage induced changes in the photoelastic coefficient ${P}_{12}$ increase non-linearly with vacancy concentration. The absolute value of the photoelastic coefficient ${P}_{12}$ decreases in damaged GaAs. Its depth profile is broader than the depth distribution of defects as predicted by the TRIM code. This indicates that the optical damage extends further than the structural damage, which is similar to the effect of GaAs implantation with other ions^26,27^. The experimental results obtained in this work are of significant importance to the theory of the photoelasticity of disordered semiconductors as well as for the GaAs based elastooptic devices operating in harsh environments or subjected to unintended defect creation during fabrication.

## Methods

### Sample preparation

GaAs (100) sample was implanted at room temperature with 140 keV hydrogen ions at $3\times {10}^{15}$ cm^−2^ fluence and 0.85 *μ*A current. No annealing was carried out following the implantation. In order to perform time-domain Brillouin scattering, a 20 nm titanium layer was deposited using Angstrom e-beam evaporator at 2 Å/s deposition rate to serve as a transducer for CAP wave. The choice of Ti is supported by the excellent acoustic impedance matching with GaAs (8%) that suppresses acoustic reflection at their interface.

### Time-domain Brillouin scattering

Time-domain Brillouin scattering measurements were performed in a standard time-resolved pump-probe setup in reflection geometry. A Coherent Mira 900 with 150-fs pulses at 76 MHz was used as a laser source. The pump and probe beams were tuned to 880 nm with 200 mW power and 10 mW power, respectively. Angle of incidence of the probe beam was 30°. The probe wavelength is tuned to the band edge of GaAs because of high sensitivity to implantation damage^27^. Both beams were focused onto the specimen with spot diameters of 100 *μ*m and 90 *μ*m for pump and probe, respectively. The pump beam was chopped using Thorlabs optical chopper at about 3 kHz.

### Data availability statement

The datasets generated and analyzed during the current study are available from the corresponding author on reasonable request.
