# Publisher Correction: The photoelastic coefficient ${P}_{12}$ of H^+^ implanted GaAs as a function of defect density

**DOI:** 10.1038/s41598-018-29607-z

**Published:** 2018-08-07

**Authors:** Andrey Baydin, Halina Krzyzanowska, Rustam Gatamov, Joy Garnett, Norman Tolk

**Affiliations:** 1 Department of Physics and Astronomy, Vanderbilt University, Nashville, TN 37235 USA; 2 Institute of Physics, Maria Curie-Sklodowska University, Pl. M. Curie-Sklodowskiej 1, 20-031 Lublin, Poland; 3 Interdisciplinary Materials Science Program, Vanderbilt University, Nashville, TN 37235 USA; 4 Department of Life and Physical Sciences, Fisk University, Nashville, TN 37208 USA

Correction to: *Scientific Reports* 10.1038/s41598-017-14903-x, published online 09 October 2017

The original version of this Article contained errors in the title and abstract of the paper, where “${P}_{12}$” was incorrectly given as “*P*_12_”. This has now been corrected in the HTML version of the Article. The PDF version was correct at the time of publication.

